# Agreement of PROCAM and SCORE to assess cardiovascular risk in two different low risk European populations

**DOI:** 10.1016/j.pmedr.2018.11.019

**Published:** 2018-12-01

**Authors:** Michel Romanens, Thomas Szucs, Isabella Sudano, Ansgar Adams

**Affiliations:** a Vascular Risk Foundation, Olten, Switzerland; b European Center of Pharmaceutical Medicine, Basel, Switzerland; c University Heart Center, Cardiology, University Hospital Zurich, Switzerland; d BAD Gesundheitsvorsorge und Sicherheitstechnik GmbH, Bonn, Germany

**Keywords:** Primary prevention, Cardiovascular risk prediction, Risk calibration, Risk discrimination

## 1 Introduction

The quality of cardiovascular risk assessment and subsequent medical treatment for those at elevated risk is dependent on the precision of resource allocation. Several confounders may lead to unnecessary treatments, while those at elevated risk may remain untreated. Confounders occur both in calibration and discrimination. While calibration defines the threshold for individuals, which labels them as low, intermediate, high or very high subjects, discrimination describes the diagnostic accuracy of a test to detect those with future disease (Romanens et al., 2010).

Once a test for cardiovascular risk assessment, such as the Framingham algorithm, has been derived from a population over decades, accuracy in different populations and continents still has to be undertaken (external validation). External validation is however difficult to obtain because contemporary performance can be assessed only after decades of observation of the outcome variable. Substitutions are however possible, e.g. with atherosclerosis imaging of carotid or coronary arteries (Romanens et al., 2017) or by measuring cardiovascular risk at the time of a cardiovascular event (Mortensen and Falk, 2017). Other important issues are the demographic or ethnic background of individuals assessed with such risk tools or the type of occupation.

The working group on lipids and atherosclerosis (AGLA) recommends the use of cardiac or cardiovascular risk derived from PROCAM and SCORE respectively in Switzerland (Eckardstein, 2014). Based on predefined risk thresholds, a primary care subject is categorized into low, intermediate or high risk. For PROCAM, the categories are 0–9%, 10–19% and 20% or more; for SCORE, the categories are 0.0–0.9%, 1.0–4.9% and 5% or more (Piepoli et al., 2016). A major limitation to calculate global cardiovascular risk is present when single cardiovascular risk factors are high. Therefore, subjects with LDL above 5.0 mmol/l or systolic blood pressure above 160 mmHg are by definition high risk in Switzerland (for SCORE, the high-risk cutoffs are 8.0 mmol/l for total cholesterol and systolic blood pressure of 180 mmHg or more).

The SCORE model based the risk algorithm on observations of fatal cardiovascular events in 12 European cohorts undergoing baseline examination between 1967 and 1991 (Conroy et al., 2003). In contrast, PROCAM was derived from working men, later extended to women using observations for myocardial infarction only (Assmann et al., 2007). Therefore, the accuracy of these risk assessment tools may be different in various populations.

For the purpose of this study, we assess the agreement for a statin indication in German and Swiss subjects using the PROCAM/AGLA and the SCORE algorithm at various calibration thresholds.

## 2 Methods

### 2.1 Subject selection

Subjects were assessed at the practice based level as described elsewhere (Romanens et al., 2014). In the Swiss Center in Olten, subjects were referred to a cardiological workup by their primary care physician (59%) or self-referred after public advertisements for a free of charge risk cardiovascular estimate payed for by to the vascular risk foundation (41%). In Koblenz, all subjects were referred within a working medicine setting (Adams and Bojara, 2015): subjects came from enterprises of different industries (chemistry, glass, pharmacy, administration, metal, social institution, paper, printing, ceramics, computer science) and 44% were employees, 56% were workman (43% of workman work in 3-shift with night shift). Subjects had to be free of cardiovascular symptoms, disease or diabetes mellitus. Laboratory values (cholesterol, LDL, HDL, triglycerides, fasting glucose), blood pressure and medical history were measured locally and entered into a spread-sheet (Excel, Microsoft, Richmond, USA). The recruitment period was between 2002 and 2017 in the Olten area and between 2011 and 2017 in the Koblenz area without a change in the standards of assessment.

### 2.2 Computation of cardiovascular risk

Cardiovascular risk was computed using the published risk formulae in an Excel spread sheet. We used the European Society of Cardiology risk calculators for low risk populations (SCORE (Descamps et al., 2012)) and the German PROCAM risk (Assmann et al., 2007). For Switzerland, PROCAM risk was multiplied by the factor 0.7 (according to the Swiss AGLA guidelines 2014 (Eckardstein, 2014)) in order to calculate the AGLA risk score. For Switzerland, it is recommended that an AGLA risk below 10% should be viewed as a low coronary risk. An individual data plot of AGLA versus SCORE was created for the Olten area.

### 2.3 Computation of statin indications

We computed a statin indication as present as follows:

A) “AGLA/PROCAM risk 10%–19 % and LDL 3.0–4.9 mmol/l” or “risk ≥ 20% and LDL 2.5–4.9 and BP < 160 mmHg” or “AGLA/PROCAM BP ≥ 160 mmHg and LDL ≥ 2.5 mmol/l” or “AGLA/PROCAM BP < 160 mmHg and LDL ≥ 5.0 mmol/l”.
B) “SCORE risk 1.0%–4.9 % and LDL 3.0–4.9 mmol/l” or “risk 1.0–4.9 % and LDL 4.0–4.9 mmol/l” or “risk ≥ 5.0 % and LDL 2.5–4.9 mmol/l and BP < 180 mmHg” or “SCORE BP ≥ 180 mmHg and LDL ≥ 2.5 mmol/l” or “SCORE BP < 180 mmHg and LDL ≥ 5.0 mmol/l”.

### 2.4 Ethical aspects

Subjects with self-referral to the Vascular Risk Foundation gave written consent. The study protocol was approved by the local ethical committee of Solothurn, Switzerland. Practice based subjects were entered into an anonymized study registry, for which current legislation in Switzerland and Germany does not require formal ethical committee consent.

### 2.5 Statistics

We used MedCalc software (Version 17.6) to calculate ROC analysis and weighted Kappa statistics (MedCalc Software bvba, 2017). Level of statistical significance was set at *p* < 0.05.

## 3 Results

### 3.1 Patient characteristics

We assessed 4,588 healthy German and Swiss subjects aged 40–65 years (Adams et al., n.d.). Clinical information for Swiss (CH) subjects from the Olten area (*N* = 1,858) was collected and compared to 2,730 German (DE) subjects from the Koblenz area. CH subjects were older (55 ± 7 versus 50 ± 6 years) with comparable results for the number of females (46% and 39% respectively) and current smokers in each group, for systolic blood pressure, lipids, and global risk scores (Table 1). The time range and the median time of assessment was between 2002 and 2017 in the Olten area (median year 2009) and was between 2008 and 2017 in the Koblenz area (median year 2012).

**Table 1:** Patient characteristics assessed between 2002 and 2017.

Population characteristics	Olten area	Koblenz area
N=	1858	2730
Female (%)	850 (46%)	1070 (39%)
Age (±SD)	55 ± 7	50 ± 6
Current smoker (N)	428 (23%)	608 (22%)
Family history^a^ (N)	358 (19%)	615 (23%)
Cholesterol (mmol/l)	5.9 ± 1.1	6.0 ± 1.0
Cholesterol ≥8.0 mmol/l (N)	76 (4%)	104 (4%)
LDL (mmol/l)	3.7 ± 1.0	3.9 ± 0.9
LDL ≥ 5.0 mmol/l (N)	194 (10%)	278 (10%)
Blood pressure (SD)	128 ± 15	125 ± 16
BP ≥ 180 mmHg (N)	8 (0%)	25 (1%)
PROCAM risk (SD)	5.5 ± 6.5	4.9 ± 6.3
AGLA risk (SD)	3.8 ± 4.5	–
SCORE risk (SD)	1.8 ± 1.7	1.3 ± 1.5

a For the occurrence of myocardial infarction or stroke in first relatives aged <60 years.

### 3.2 Distribution of risk categories and potential for statin indication

In the Olten area, AGLA categorizes 89% as low risk (SCORE 38%), in the Koblenz area PROCAM and SCORE classification was 83% and 56% respectively. Intermediate risk was found in 10% with AGLA and in 56% with SCORE (Koblenz: 12% und 41% respectively). High risk was rare with AGLA (2%) and SCORE with 6% (Koblenz: 4% and 3% respectively, Table 2). Agreement for risk categories using linear weighted Kappa statistics was poor in the Olten area between AGLA and SCORE (weighted Kappa 0.152, 95% CI: 0.127 to 0.177), significantly lower than in the Koblenz area but remained weak (weighted Kappa 0.385, 95% CI: 0.355 to 0.416, Table 3a, Table 3b).

**Table 2:** distribution of risk categories (in percent) assessed between 2002 and 2017.

	Olten area		Koblenz area	
	AGLA	SCORE	PROCAM	SCORE
Low (L)	89%	38%	83%	56%
Intermediate (M)	10%	56%	12%	41%
High (H)	2%	6%	4%	3%

**Table 3a:** Agreement (weighted kappa statistics) between risk categories for the Olten area utilizing AGLA and SCORE assessed between 2002 and 2017.

		SCORE		
		L	M	H
AGLA	L	551	697	41
	M	1	104	35
	H	0	11	14

Weighted Kappa 0.152 (95% confidence interval: 0.127 to 0.177).

**Table 3b:** Agreement (weighted kappa statistics) between risk categories for the Koblenz area utilizing PROCAM and SCORE assessed between 2002 and 2017.

		SCORE		
		L	M	H
PROCAM	L	1292	643	10
	M	12	254	24
	H	0	66	36

Weighted Kappa 0.385 (95% confidence interval: 0.355 to 0.416).

**Fig. 1:**
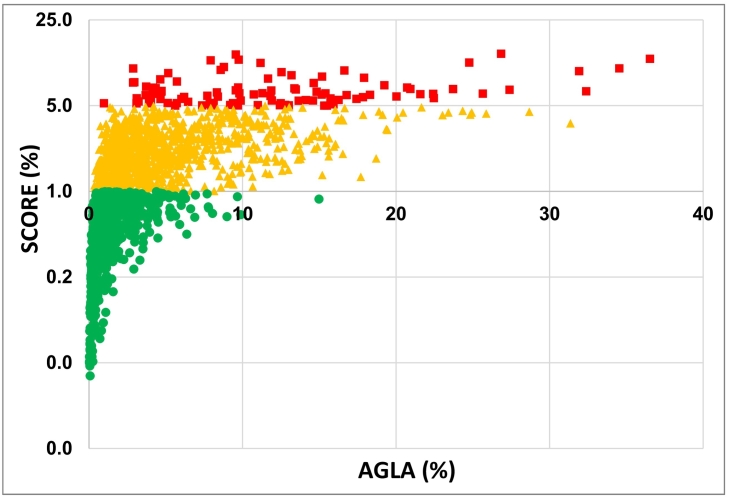
Individual data plot of AGLA (horizontal axis) and SCORE (logarithmic scale, vertical axis) for 10-year risk estimates in percent assessed between 2002 and 2017. Green color denotes low SCORE risk (<1.0%), yellow denotes intermediate SCORE risk (1.0–4.9%) and red denotes high SCORE risk (≥5%). (For interpretation of the references to color in this figure legend, the reader is referred to the web version of this article.)

A scatter plot of the Olten area subjects (Fig. 1) shows the distribution of AGLA versus SCORE across all risk categories.

We found a statin indication based on cardiovascular risk results using AGLA/PROCAM in 5% in the Olten region and in 9% in the Koblenz region. Another 13% qualified for statins because of extreme blood pressure or LDL values in the Olten region (14% in the Koblenz region). In contrast, using the SCORE calculator, a statin indication was present in 40% for LDL cutoff between 3.0 and 5.0 mmol/l (19% for the LDL cutoff between 4.0 and 4.9 mmol/l) in Switzerland (30% and 17% in the Koblenz region, respectively, Table 4). In total, 18% qualified for a statin in the Olten region using AGLA (PROCAM 22%) and for SCORE we found a total indication for statins in 51% in the Olten region (LDL cutoff 3.0–4.9 mmol/l).

**Table 4:** Indication for statin or intensified statin treatment for AGLA/PROCAM and SCORE assessed between 2002 and 2017.

	Olten area		Koblenz area	
Definition for statin indication	(N)	(%)	(N)	(%)
AGLA/PROCAM 10–19% and LDL 3.0–4.9 mmol/l (N, %)	61	3%	184	7%
AGLA/PROCAM ≥20% and LDL 2.5–4.9 and BP < 160 mmHg (N, %)	40	2%	50	2%
AGLA/PROCAM BP ≥ 160 mmHg and LDL ≥ 2.5 mmol/l	50	3%	106	4%
AGLA/PROCAM BP < 160 mmHg and LDL ≥ 5.0 mmol/l	189	10%	260	10%
High risk only AGLA PROCAM	279	15%	416	15%

Sum of AGLA/PROCAM	340	18%	600	22%
SCORE 1.0–4.9% and LDL 3.0–4.9 mmol/l (N, %)	682	37%	769	28%
SCORE 1.0–4.9% and LDL 4.0–4.9 mmol/l (N, %)	292	16%	401	15%
SCORE ≥ 5.0% and LDL 2.5–4.9 mmol/l and BP < 180 mmHg (N, %)	62	3%	49	2%
SCORE BP ≥ 180 mmHg and LDL ≥ 2.5 mmol/l	7	0%	24	1%
SCORE BP < 180 mmHg and LDL ≥ 5.0 mmol/l	194	10%	274	10%
High risk only SCORE	263	14%	347	13%

SUM of SCORE LDL cutoff 3.0–4.9 mmol/l in	945	51%	1116	41%
SUM of SCORE LDL cutoff 4.0–4.9 mmol/l in	555	30%	748	27%

Note: for all patients (*N* = 4588) and LDL cutoff for statin indication of ≥3.0 mmol/l wKappa was 0.454 (95% CI: 0.431–0.477) with a disagreement in 26% of cases (25% would be treated with SCORE, but not with AGLA/PROCAM) at the LDL 3.0 mmol/l cutoff.

## 4 Discussion

The Swiss working group on lipids and atherosclerosis (AGLA) recommends to use cardiac risk or cardiovascular risk derived from PROCAM and SCORE respectively in Switzerland (Eckardstein, 2014). In this study, we assessed the agreement for the risk categories “low”, “intermediate” and “high” for both risk calculators in two large independent populations from the Olten area in Switzerland and the Koblenz area in Germany and discuss important limitations about calibration and discrimination when using contemporary risk assessment tools at the population and at the individual level.

We extend and confirm a previous study from the Olten area published in 2005 with a wKappa value of 0.22 (Romanens et al., 2009) and another study from the Lausanne area with again a wKappa value of 0.22 (Riesen et al., 2005) regarding the agreement of risk categories. The reason for differences between the Olten and the Koblenz area cannot, however, be elucidated from our data. Fig. 1 gives an impression of a plot of SCORE and AGLA in the Olten area at the individual level, where it becomes apparent, that many subjects with AGLA risk below 10% are at intermediate or even at high risk with SCORE. From this it can be recommended to calculate SCORE in those with AGLA <10%. However, both AGLA and SCORE were not validated externally for Switzerland in prospective cohorts.

Most primary care physicians use the AGLA calculator and its inherent statin recommendations and may therefore miss up to 72% of the statin indications given by the presence of pronounced risk factors as outlined in Table 4.

In 2005, where SCORE recommended statins only in high risk patients (≥5.0%), we found a statin indication in 713 subjects for AGLA in 19% and for SCORE in 6% (Romanens et al., 2009). Similarly, using a group of 8,829 subjects from the Lausanne Health Promotion Program (Prior et al., 2005), a potential statin indication was found for AGLA in 23% and for SCORE in 2% (Riesen et al., 2005). Compared to 2005, statin indications based on guidelines revealed stable numbers for AGLA/PROCAM (around 20%) and substantially increased for SCORE from about 2%–6% to 30%–40% for the LDL cutoff 3.0 mmol/l. If a more restrictive use of the statin indication is followed using the European Guidelines on cardiovascular disease prevention in clinical practice (Piepoli et al., 2016), where statins should “at least be considered” only in SCORE risk ≥5.0% and an LDL ≥ 4.0 mmol/l, then 17%–19% would have a statin indication. If statins should “at least be considered” only in AGLA/PROCAM risk ≥20% and or LDL ≥ 5.0 mmol/l, then a statin indication would be present in 12% in both the Olten area as well as the Koblenz area (Table 4). The poor agreement regarding risk categories and the only moderate agreement regarding statin indications are clinically disturbing. While the recommendations for treatment cutoffs in arterial hypertension are clear cut (Mancia et al., 2013), the recommendations about statins are confusing, especially when reading the European guidelines (Piepoli et al., 2016): “Thus treatment may *occasionally be considered in moderate risk* (1–5%) individuals, provided that patients are well-informed of the limited absolute risk reduction, and high numbers needed to treat. In higher risk (5–10%), drug therapy is associated with somewhat larger absolute benefits, and should at least be considered.” This wording reflects a substantial uncertainty regarding the indication for statins in primary care and may lead to poor agreement regarding statin indications (Romanens et al., 2017; Mortensen and Falk, 2017).

Both AGLA and SCORE have been assessed with respect to the risk category in a cross-sectional study of 3,172 adults without previous CVD hospitalized with acute coronary syndromes (ACS) at 4 university centers in Switzerland (Selby et al., 2015). In those ACS patients without diabetes, AGLA categorized them into low risk in 58%, into medium risk in 18% and into high risk in 24% (SCORE 36%, 38%, 26% respectively). Therefore, most ACS patients are categorized into the low or intermediate risk group, especially prominent for the AGLA risk calculator. Importantly, of the 3,172 ACS patients, only 16% were treated with statins at the time of the event, while 69% had a statin indication based on the ESC primary prevention targets and 55% had a statin indication with the AGLA 2012 recommendation.

According to the European Recommendations, the presence of carotid plaque is viewed as a *very high risk* finding (≥10% cardiovascular mortality risk in 10 years) (Piepoli et al., 2016). We have published the amount of carotid plaque using a surface tracing technique (total plaque area, TPA (Spence et al., 2002)) in 2,202 healthy subjects from the Olten area and in 2,942 healthy subjects from the Koblenz area (Romanens et al., 2017). The prevalence of advanced atherosclerosis (TPA ≥ 80 mm^2^) in middle-aged subjects (40–55 years) was 11% in the Olten area and was 13% in the Koblenz area. The sensitivity of AGLA in the Olten area at the 10% threshold was 0% for women and 10% for men (SCORE at the 1% threshold was 28% and 68%, at the SCORE threshold of 5% was 0% and 3% respectively). The sensitivity of PROCAM at the 10% threshold was 4% for women and 45% for men from the Koblenz area (SCORE at the 1% threshold was 15% and 76%, at the SCORE threshold of 5% was 0% and 4% respectively (Romanens et al., 2017), Supplementary tables). Further, we found the area under the curve to detect TPA ≥ 80 mm^2^ to be significantly lower for AGLA when compared to SCORE in the Olten area (74.3% versus 77.3%, *p* = 0.003). Based on these observations, the use of SCORE appears to be a safer strategy in primary prevention than the use of AGLA both with respect to calibration and discrimination if a SCORE threshold of 1.0%–4.9% is used for intermediate risk. The low sensitivity for clinical cardiovascular events and extensive atherosclerosis raises questions regarding the calibration factor of AGLA, which is PROCAM multiplied by 0.7. In view of the clinically relevant sensitivity problem of AGLA, the reduction of myocardial infarction risk by 30% for Switzerland when compared to Germany needs further validation. Calibration factors have been proposed for several areas, e.g. Strasbourg (women: 0.88, men 0.90), for ex-Yugoslavia (women 1.24, men 1.37) or for Poland (women 2.22, men 2.21) (Romanens and Ackermann, 2006). It may therefore be prudent, not to use the current calibration factor of 0.7 in various ethnic groups living in Switzerland. Poorly performing calibration factors (low sensitivity for atherosclerosis and outcome) will unnecessarily delay timely preventive interventions, for instance with statins. This will lead to a substantial increase in long-term morbidity and all-cause mortality as has recently been shown for the principle of compression of morbidity in the Chicago area (Allen et al., 2017). An important difference between AGLA/PROCAM and SCORE is the outcome variable estimate, which is myocardial infarction for AGLA/PROCAM (CHD) and which is myocardial infarction, stroke and coronary revascularisation for SCORE (CVD). According to the numbers of the Cholesterol Treatment Trialist (CTT) metaanalysis, for every myocardial infarction (17.6% observed in 5 years), another 8% develop a stroke and another 15.8% need a coronary revascularisation procedure (Mihaylova et al., 2012). Therefore, CHD risk would have to be multiplied by a factor 2.35 in order to estimate CVD risk from CHD risk. If we look at treatment costs in 2011 in Switzerland for myocardial infarction (4,780 million Swiss francs) and compare it with the treatment costs for stroke (3,170 million Swiss francs), then the rationale for estimates that include stroke risk becomes evident (Wieser et al., 2014). As a limitation, the long collection time of data in the Olten area (2002–2017) might have affected our results.

## 5 Conclusion

Cardiovascular risk assessment, single risk factors and atherosclerosis imaging have all limitations with respect to sensitivity and specificity for future cardiovascular events. Nevertheless, efforts are to be made in order to improve risk prediction. Assessing calibration and discrimination in subjects with an acute coronary event may help to adjust risk thresholds. In the future, atherosclerosis imaging (coronary calcifications, significant amounts of carotid plaque) may help to adjust for poor discrimination and may help to prefer one algorithm over the other within the observed population or calibration factors may be adopted according to the ethnical background, psychological or work load stress.

## Funding sources

This research did not receive any specific grant from funding agencies in the public, commercial, or not-for-profit sectors.

## Conflict of Interest and compliance with ethical standards

The authors declare that there is no conflict of interest.

## Ethical approval

All procedures performed in studies involving human participants were in accordance with the ethical standards of the institutional and/or national research committee and with the 1964 Helsinki declaration and its later amendments or comparable ethical standards.
